# Perforated Meckel’s Diverticulum Caused by a Toothpick: A Case Report and Review of Literature

**DOI:** 10.7759/cureus.37390

**Published:** 2023-04-10

**Authors:** Tatiana Fernandez Trokhimtchouk, Luis F Flores, Álvaro Morillo Cox, Estefanie S Otanez, Joseline K Crespo Martinez

**Affiliations:** 1 General Surgery, Univesidad Internacional del Ecuador, Axxis Hospital, Quito, ECU

**Keywords:** case report, intestinal perforation, laparotomy, meckel’s diverticulum, acute abdomen

## Abstract

Although Meckel's diverticulum is the most common congenital anomaly of the gastrointestinal tract, it is rare in the general adult population. When it does become symptomatic, it is usually due to complications such as perforation. We report the case of a 38-year-old man who presented with acute abdominal pain in the right iliac fossa, fever, and tachycardia. Complementary exams at the emergency department showed leukocytosis and elevated C-reactive protein. Acute appendicitis was suspected, so he was taken to the operating room for a diagnostic laparoscopy. During surgical exploration, a perforated Meckel’s diverticulum caused by a toothpick was found. Surgery was converted to laparotomy with resection of the small bowel segment containing the diverticulum, followed by a primary anastomosis. The postoperative period was uneventful, and the patient was discharged on day seven. No abnormalities were reported in the histopathology study. In this report, we review and discuss similar cases found in the literature, all of them male with acute abdomen and suspicion of appendicitis. We aim to remark on the importance of keeping in the differential of such patients a perforated Meckel’s diverticulum.

## Introduction

Acute abdomen is a common presenting problem in the emergency department and includes a wide range of differential diagnoses. Meckel's diverticulum is a rare complication that can cause symptoms. Although it is the most common congenital anomaly of the gastrointestinal tract, it is only present in 2%-3% of the population [1]. It is typically discovered incidentally during surgical procedures and has a 4% lifetime rate of complications, with males being the most commonly affected. Obstruction is the most common complication in adults at a rate of 36.5%, followed by diverticulitis, perforation, and hemorrhage [2]. Perforation by a foreign body is rare, with only a few cases reported in the literature. A variety of items are found, i.e., toothpicks, fish bones, chicken bones, etc., and there seems to be a predisposition for foreign bodies to embed themselves in the diverticulum. The management of symptomatic Meckel’s diverticulum is surgical, but there is controversy over how to proceed when it is found incidentally during abdominal exploration [3].

We report the case of a male adult who presented to the emergency department complaining of abdominal pain and was found to have a perforated Meckel's diverticulum by a toothpick during surgical exploration.

We reviewed the literature in search of similar cases and found five such reports. All of them were men, initially suspected to have appendicitis. It is important to consider this differential in the evaluation of acute abdomen.

## Case presentation

The patient, a 38-year-old man with no medical or surgical history, was admitted to the emergency department complaining of abdominal pain that started 48 hours prior and intensified over 18 hours before without an identifiable cause. The pain was initially diffuse but later became localized to the right iliac fossa and was accompanied by nausea, vomiting, and fever.

His vital signs showed mild tachycardia and fever. His physical exam was remarkable for a tense abdomen; bowel sounds were abolished; and the pain was exerted on light palpation diffusely, with marked peritoneal reaction on the right iliac fossa.

His blood workup showed leukocytosis of 18.000/mm3 with 85% neutrophils, a C-reactive protein of 154 mg/L, creatinine of 1.32 mg/dL, a glomerular filtration rate of 71 ml/min/1.73 m2, hyperbilirubinemia of 2.54 mg/dL, and an unconjugated fraction of 1.94 mg/dL. Liver enzyme tests were within normal ranges. An abdominal ultrasound was performed, which showed no apparent signs of pathology and was inconclusive.

**Figure 1:**
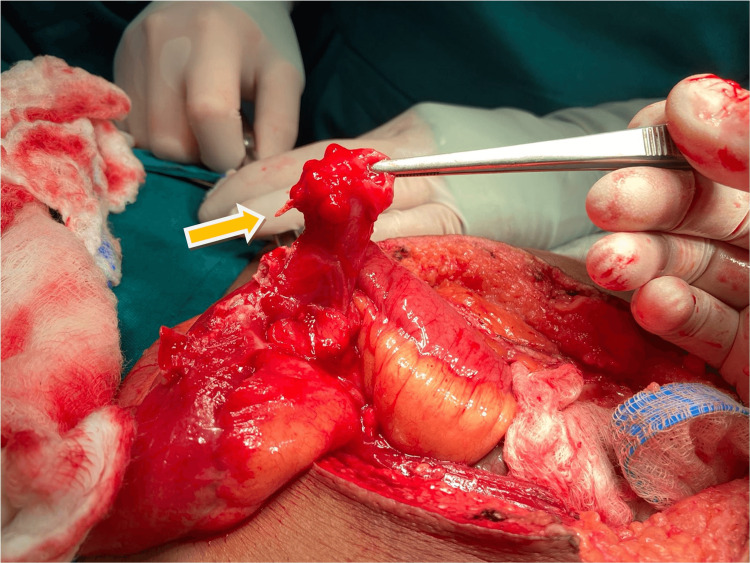
A transoperatory photograph showing a Meckel’s diverticulum perforated by a toothpick (yellow arrow)

Under the suspicion of an acute abdomen of probable appendicular etiology, he was taken to the operating room for an urgent diagnostic laparoscopy. Upon initial exploration, a normal appendix was found, along with a phlegmon adhered to the anterior abdominal wall. The procedure was converted to a laparotomy by a midline incision. After the dissection of the phlegmon, a Meckel’s diverticulum was found 50 cm proximal to the ileocecal valve. It was perforated by a foreign body, a toothpick, as shown in Figure 1.

**Figure 2:**
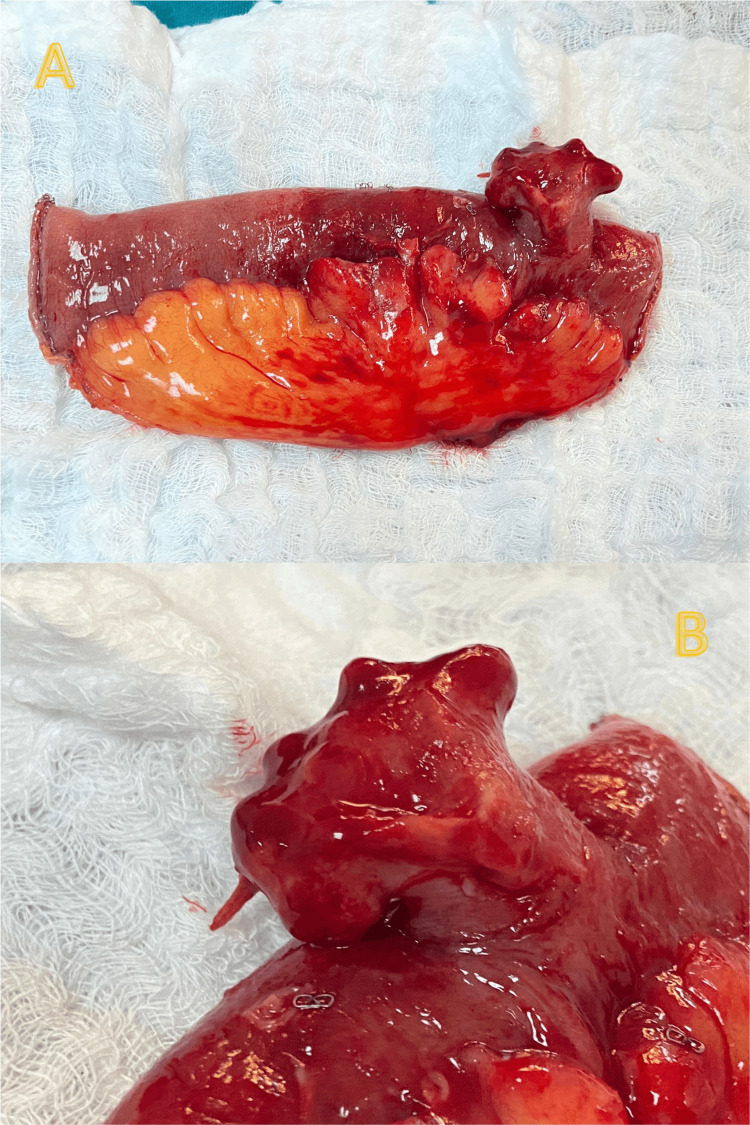
Surgical specimen. (A) The resected portion of the small bowel containing the Meckel’s diverticulum; (B) shows a close-up

The resection of the compromised small bowel segment was performed with a mechanical latero-lateral primary anastomosis. The specimen is shown in Figure 2. An incidental appendectomy was done as well. A closed-suction drain was left in place.

He was started on intravenous ceftriaxone and metronidazole before surgery and continued postoperatively; both were given for five days. He started oral intake on postoperative day (POD) two with adequate tolerance and was discharged home on POD seven. The drain was removed before discharge. The histopathology report confirmed the findings, showing a mural acute inflammatory gangrenous process associated with the perforation; no aberrant tissue was found within the diverticulum.

## Discussion

Acute abdomen is a condition that needs urgent surgical evaluation. It can be caused by many reasons, including infection, inflammation, vascular occlusion, or intestinal obstruction. The clinical picture comprises a wide variety of differential diagnoses to be considered, one of them being a complicated Meckel’s diverticulum. Although rare, the physician must always bear the diagnosis in mind.

As described, Meckel’s diverticulum is the most common congenital anomaly of the gastrointestinal (GI) tract, caused by incomplete closure of the omphalomesenteric duct and affecting 2%-3% of the population. It is estimated that the diverticulum becomes symptomatic in 4%-16% of the individuals, with a predominance in males during childhood, presenting most often as lower GI bleeding [4, 5]. Complications can include obstruction, diverticulitis, hemorrhage, and perforation, the latter being rare.

A small percentage of foreign bodies may lodge in the Meckel’s diverticulum and cause perforation, leading to an acute abdomen and requiring urgent surgical exploration. There are reports of multiple items that can cause perforation, such as fish bones, dentures, toothpicks, chicken bones, needles, batteries, and other small sharp objects. While foreign bodies can be ingested accidentally, there have also been cases where children have swallowed small objects intentionally.

The diagnosis of perforated Meckel’s diverticulum is usually done during surgical exploration due to the symptoms being non-specific, as it often mimics acute appendicitis. It is also stated that less than 10% of symptomatic cases are diagnosed preoperatively [6].

We present the case of an adult male who was diagnosed with a perforated Meckel's diverticulum caused by a toothpick during surgery after being initially suspected to have acute appendicitis, which was ruled out with intraoperative findings. The patient had no recollection of ingesting the foreign body. Although rare, similar cases have been reported in the literature. Zingg et al. reported a case of an adult male who presented with right lower quadrant pain without peritoneal signs and was found to have a perforated Meckel's diverticulum by a toothpick during laparoscopy [7]. Kadhi et al. described the case of a 13-year-old boy with signs of acute appendicitis who was diagnosed with a perforated Meckel's diverticulum caused by a wooden toothpick that had pierced through the tip of the diverticulum during laparoscopy [8]. Other cases reported by Naufel C., Wang et al., and Greenspan et al. also presented similar clinical pictures, with all patients being male, suspected to have appendicitis, and diagnosed during surgery of a perforated Meckel's diverticulum by a toothpick [9, 10, 11].

Despite the fact that Meckel’s complication is uncommon, it should be considered a differential diagnosis when a patient presents with right iliac fossa pain and an acute abdomen. In all the reported cases, the findings were made during surgery for suspected appendicitis and not preoperatively. We report this case with the aim of suggesting to always consider in the differential diagnosis a complicated Meckel’s diverticulum when evaluating a patient with an acute abdomen and suggestive appendicitis.

## Conclusions

Abdominal pain is a common reason for emergency consultations, and an acute abdomen requires urgent surgical evaluation. Although rare, perforation of a Meckel’s diverticulum by a toothpick can mimic acute appendicitis. A diagnosis is typically made during surgery due to the non-specific presentation and inconclusive imaging findings. Management options may include diverticulectomy and resection of the small bowel segment with primary anastomosis. It is essential to consider this differential diagnosis when evaluating acute abdomen cases.
